# Bidirectional Associations between Obesity and Cognitive Function in Midlife Adults: A Longitudinal Study

**DOI:** 10.3390/nu11102343

**Published:** 2019-10-02

**Authors:** Andree Hartanto, Jose C. Yong, Wei Xing Toh

**Affiliations:** 1 School of Social Sciences, Singapore Management University, Singapore 178903, Singapore; 2 Department of Psychology, National University of Singapore, Singapore 117570, Singapore

**Keywords:** obesity, body mass index, waist-to-hip ratio, executive function, episodic memory

## Abstract

The links between obesity and cognition remain equivocal due to a variety of methodological limitations with current research, such as an overreliance on body mass index (BMI) as a measure of obesity, the use of cross-sectional designs, and inadequate specification over the domains of cognitive function to be examined. To address these issues, we used data from the Cognitive Project of the National Survey of Midlife Development in the United States, a large-scale, longitudinal dataset on non-institutionalized midlife adults (*N* = 2652), which enabled us to examine the long-term bidirectional relations between obesity and two latent factors of cognition—executive function and episodic memory—while controlling for potential confounds. Results showed that, over a span of nine years, an increase in obesity in Time 1 is associated with a decline in episodic memory in Time 2 (but not executive function), while an increase in executive function in Time 1 (but not episodic memory) is associated with a reduction in obesity in Time 2. These results were elucidated when obesity was indexed with waist-to-hip ratio but not with BMI. Our findings highlight important directions for further research, in particular the use of more valid obesity indices and a greater focus on the bidirectional effects between obesity and cognition.

## 1. Introduction

Obesity is a widely recognized risk factor for a variety of chronic physical health ailments including metabolic syndrome, hypertension, cardiovascular disease, diabetes, stroke, and cancer [1]. Less known, however, is the impact of obesity on cognitive functioning. On the one hand, excess adiposity has been implicated in neurocognitive decline, with some studies finding obesity to be associated with reduced cognitive ability [2,3,4], increased risk of dementia [5,6], and structural brain changes such as excess age-related atrophy and white matter disease [7,8,9]. On the other hand, findings on the cognitively detrimental effects of obesity appear to be mixed. For example, some recent studies failed to find evidence that obesity increases dementia risk [10,11], while others even found underweight individuals to be at greater risk instead, thereby counterintuitively presenting obesity as a protective factor [12,13].

Several methodological factors may account for these mixed findings. First, the influence of obesity on cognitive function may be obscured by the popular use of body mass index (BMI) as a measure of obesity, which has been increasingly shown to be suboptimal to alternative measures such as waist-to-hip ratio (WHR) [14]. For instance, in one large sample of midlife adults, WHR (but not BMI) was linked to cognitive deficits over and above the influence of demographics, health factors, personality, and self-perceived obesity [15]. Second, an overreliance on cross-sectional designs also limits our conclusions to static associations between obesity and cognitive functioning at single time points [16], and most studies neglect the possible bidirectional effects between obesity and cognition [17,18]. On the one hand, impaired cognition may hinder people’s ability to regulate weight [19,20]; on the other hand, obesity may produce neurological changes [21,22,23] that underlie reduced motivation, self-control, and cognitive ability [24]. Lastly, the impact of obesity on cognition appears to be domain specific. For instance, obesity has been documented to impair more memory-related cognitive functions, such as episodic memory, rather than executive functions [25].

To address these methodological gaps and establish greater precision over the links between obesity and cognition, the present study sought to investigate the bidirectional relationships between obesity and two key domains of cognitive ability, specifically executive functions and episodic memory, using a large-scale, longitudinal dataset on midlife adults. In so doing, we simultaneously shed light on the bidirectional nature and domain-specificity of the obesity–cognition relationship as well as how this relationship unfolds over time. To achieve these objectives, we draw from the Cognitive Project of the National Survey of Midlife Development in the United States (MIDUS), which affords an invaluable opportunity to examine our relationships of interest with large, non-clinical, and representative samples over multiple time points.

## 2. Method

### Participants

The current study comprises 2683 midlife adults who participated in the Cognitive Project under the second (II) and third (III) waves of the MIDUS (Midlife Development in the United States). MIDUS II was conducted between 2004 and 2006 on a random-digit-dial sample of non-institutionalized, English-speaking adults. MIDUS II participants were aged between 33 and 84 (*M* = 55.04, *SD* = 11.21) with females making up 55% of the sample. MIDUS III was conducted between 2013 and 2017 as a follow-up to MIDUS II using the same methodology and assessments. In accordance with established exclusion criteria [25], we excluded underweight participants (BMI > 18.5) in both waves to omit effects associated with being underweight on cognition (e.g., health problems, malnourishment due to poverty, anorexia nervosa, etc.; *n* = 31), which resulted in a final sample size of 2652. Table 1 summarizes the descriptive statistics of our sample across both waves. All participants in the MIDUS project provided informed consent and data collection was approved by the Institutional Review Board at the University of Wisconsin-Madison. Data and materials of the current study can be accessed from the Inter-University Consortium for Political and Social Research website.

## 3. Measures

### 3.1. Cognitive Ability

Our focal subdomains of cognition—executive functions and episodic memory—were measured in the Cognitive Project of MIDUS II and MIDUS III with the Brief Test of Adult Cognition by Telephone (BTACT) [27]. The BTACT is a well-validated battery of cognitive function tests comprising the Immediate Word List Recall Task, Backward Digits Span, Categorical Fluency, Stop and Go Switch Task (SGST), Number Series, Backward Counting Task, and Delayed Word List Recall. Both exploratory and confirmatory factor analyses of the seven cognitive tests in the BTACT reveal that the data fits a two-factor model of executive function and episodic memory [28,29]. Executive function is best represented by performance on the Backward digit Span, Categorical Fluency, Number Series, Backward Counting, and SGST, while episodic memory is best represented by performance on the Immediate Word List Recall Task and Delayed Word List Recall.

### 3.2. Obesity

Obesity was indexed in terms of BMI and WHR. Participants’ BMI was computed with their self-reported weight and height based on the formula where BMI equals to kilograms per meters squared. Participants’ WHR was computed by taking the ratio of their waist around the navel to their hips at the wide point. To ensure the accuracy of the reported body measurements, participants were provided with a tape measure during the study and instructed to stand upright, keep the tape measure taut to the body, and measure at the level of their navel for the waist measurement and the wide point between their waist and thighs for the hip measurement. Based on the recommendations of previous studies [30], we operationalized BMI and WHR as continuous variables to minimize bias associated with treating obesity as a categorical variable.

## 4. Data Analysis

The present study aimed to examine the bidirectional longitudinal associations between obesity and two important domains underlying cognitive function: Executive functions and episodic memory. To accomplish this objective, we conducted our analyses with a two-wave cross-lagged design and structural equation modelling using maximum likelihood estimate with robust standard errors (MLR) on M*plus* version 7.4, which is robust to non-normality and non-independence of observation [31]. The cross-lagged panel models included autoregressive paths, cross-sectional paths between obesity and cognitive ability, and cross-lagged reciprocal paths between obesity and cognitive ability. Separate models were conducted on BMI and WHR to provide greater precision over the estimates of each measure of obesity [15]. In addition, both BMI and WHR were winsorized to minimize the influence of outliers. Missing data were handled with the full information maximum likelihood method, which is a superior, unbiased, and more efficient data imputation method than traditional ad hoc missing-data techniques in structural equation models [32].

Consistent with previous studies that estimated cognitive function in terms of executive functions and episodic memory [28,29], we indexed executive function by performance on the backward digit span, categorical fluency, number series, backward counting, and SGST; and episodic memory by performance on the immediate word list recall and delayed word list recall. To evaluate model fit, we followed an established criterion where acceptable fit is indicated when the root mean square error of approximation (RMSEA) is less than 0.08, Bentler’s comparative fit index (CFI) and Tucker–Lewis index (TLI) values are above 0.90, and the standardized root mean-squared residual (SRMR) is less than 0.08 [33,34]. 

Prior to specifying the structural model, we tested for measurement invariance to ensure that our constructs are conceptually consistent over time and that changes in our latent variables are not an artifact of changing measurement properties. The measurement invariance test included examinations of configural (equality in factor structure), metric (equality in factor loadings), and scalar (equality in latent intercepts) invariances [35]. To establish invariance, a change in CFI of less than 0.01 (ΔCFI < 0.01) between models with constraints added is recommended [36].

While retaining the imposed equality constraints, we estimated three structural models each with an additional set of covariates to ensure the robustness of any longitudinal relations revealed by our analyses. First, we estimated the cross-lagged model without controlling for covariates to provide unadjusted estimates of the longitudinal bidirectional associations between obesity and cognition over a span of approximately 9 years. Next, we controlled for potential demographic and socioeconomic status (SES) confounds at Time 1, including age at assessment, gender, education attainment, and household income [37,38,39,40,41]. Third, we controlled for health-related variables that are known to covary with both obesity and cognitive decline, including history of smoking, alcohol abuse, hypertension, diabetes, and stroke [42,43]. 

Lastly, we conducted exploratory analyses with executive function and episodic memory as a single latent factor of global cognitive function and explored differential associations between obesity and cognitive functions across time and age group as well as across time and gender.

## 5. Results

### 5.1. Measurement Invariance

We first tested for measurement invariance to verify the temporal stability of the conceptual and measurement properties of our latent variables (see Table 2). All the autocorrelations among measurement residuals were estimated to account for indicator-specific effects over time [44]. In addition, our factors were specified to have nondirectional covariance relationships. Our analyses showed that the data fitted the configural, metric, and scalar invariance models well (RMSEAs < 0.08, CFIs > 0.90., TLIs > 0.90, and SRMRs < 0.08). Full invariance of the BTACT was supported in our configural and metric models (ΔCFIs ≤ 0.001) but not in the scalar model (ΔCFI ≥ 0.027). Thus, as recommended by Byrne, Shavelson, and Muthén [45], we tested for partial scalar invariance on the basis of full metric invariance by relaxing the constraints on the intercept of the performance on SGST. The modified model fitted the data well, thus confirming partial scalar invariance for the BTACT (ΔCFI = 0.001). Given that the cross-lagged panel model is robust to minor violations of scalar invariance when the majority of our indicators are established as invariant [44], we proceeded with our analyses. In our subsequent structural models, we retained the optimal equality constraints (i.e., partial scalar invariance) to ensure that the measurement properties of our latent variables are stable over time and that the changes in these latent variables are not a byproduct of changes in measurement properties. 

### 5.2. Waist-To-Hip Ratio

**Figure 1:**
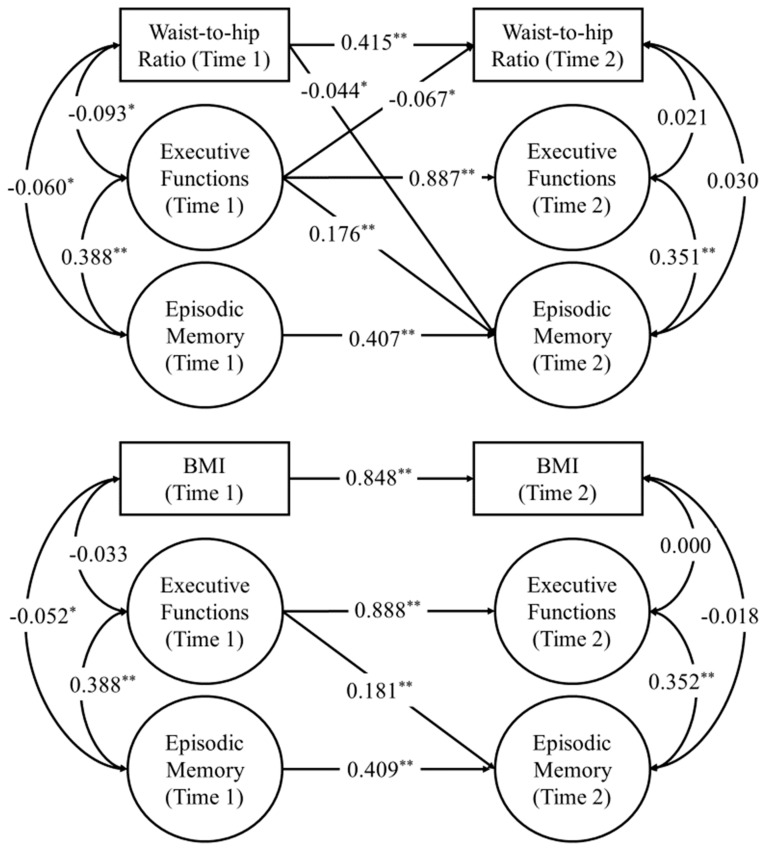
Cross−lagged panel models after controlling for age, sex, education attainment, and household income. For the purposes of clarity, factor indicators, autocorrelations among indicator residuals, and control variables are not shown, and only significant cross−lagged paths are displayed. The analyses were conducted while holding the optimal equality constraints (partial scalar invariance). The numbers are standardized coefficient estimates. * *p* < 0.05 ** *p* < 0.001.

With the quality constraints held, we conducted cross-lagged panel models to examine the bidirectional longitudinal relations between WHR and cognitive ability. All lagged paths were converted into directional predictive paths. The autoregressive and cross-lagged paths across all three structural models are summarized in Table 3. We found that WHR at Time 1 significantly predicted a negative change in episodic memory at Time 2 in the unadjusted model (β = −0.125, *SE* = 0.017, 95% CI [−0.153, −0.097], *p* < 0.001). Importantly, the cross-lagged path of WHR at Time 1 on episodic memory at Time 2 remained significant even after controlling for demographics and SES covariates (β = −0.045, *SE* = 0.019, 95% CI [−0.078, −0.013], *p* = 0.020) as well as health covariates (β = −0.044, *SE* = 0.019, 95% CI [−0.076, −0.013], *p* = 0.022), thus suggesting that an increase in WHR is uniquely associated with a higher rate of episodic memory decline after nine years. However, we did not find evidence that WHR at Time 1 predicted changes in executive function at Time 2 in any of our structural models (*p*s > 0.05). In contrast, we found that executive function at Time 1 significantly predicted reductions in WHR at Time 2 after controlling for demographics and SES (β = −0.073, *SE* = 0.034, 95% CI [−0.128, −0.018], *p* = 0.030) and health covariates (β = −0.067, *SE* = 0.034, 95% CI [−0.123, −0.011], *p* = 0.048), suggesting that executive function was uniquely associated with higher rate of decrease in WHR (See Figure 1). Lastly, episodic memory at Time 1 was not significantly associated with WHR at Time 2 (*p*s > 0.05) 

### 5.3. Body Mass Index

Similar structural models were estimated to examine the bidirectional longitudinal relations between BMI and cognitive ability. As shown in Table 3, our analyses did not reveal any cross−lagged relations between BMI and our latent factors of cognitive ability, specifically executive function and episodic memory. In contrast to WHR, BMI at Time 1 did not predict any changes in episodic memory in the unadjusted cross−lagged panel model (β = 0.015, *SE* = 0.019, 95% CI [−0.016, 0.046], *p* = 0.426), the cross-lagged panel model with demographic and SES covariates (β = 0.007, *SE* = 0.018, 95% CI [−0.022, 0.037], *p* = 0.683), or the cross−lagged panel model with demographic, SES, and health covariates (β = 0.005, *SE* = 0.019, 95% CI [−0.026, 0.035], *p* = 0.805). Likewise, neither did executive function nor episodic memory at Time 1 predict changes in BMI at Time 2 across all structural models (*p*s > 0.05). 

### 5.4. Global Cognitive Function

We further analyzed our data with executive function and episodic memory as a single latent factor of global cognitive function, which is indicated by performance on the backward digit span, categorical fluency, number series, backward counting, SGST, immediate word list recall, and delayed word list recall. Based on our modification indices, we allowed the error terms between immediate word list recall and delayed word list recall within and between two time points to be correlated as both tasks implicate highly overlapping cognitive abilities. As shown in Table 4, after the inclusion of the residual correlation between immediate word list recall and delayed word list recall, our data fitted the configural, metric, and scalar invariance models well (RMSEAs < 0.08, CFIs > 0.90, TLIs > 0.90, and SRMRs < 0.08). Full invariance was supported in our configural and metric models (ΔCFIs ≤ 0.001) but not in the scalar model (ΔCFI ≥ 0.026). After relaxing the constraints on the intercept of the performance on SGST, partial scalar invariance was achieved (ΔCFI = 0.001). 

With the equality constraints held, we conducted cross-lagged panel model analyses to examine the bidirectional longitudinal relations between obesity (WHR and BMI) and global cognitive function. As shown in Table 5, our structural models revealed that the latent factor of global cognitive function at Time 1 was significantly associated with WHR at Time 2 after controlling for demographics and SES (β = −0.075, *SE* = 0.030, 95% CI [−0.133, −0.016], *p* = 0.012) and health covariates (β = −0.068, *SE* = 0.030, 95% CI [−0.127, −0.009], *p* = 0.023). However, WHR at Time 1 was not associated with global cognitive function at Time 2 (*p*s > 0.05). Similarly, we did not find any evidence of bidirectional longitudinal relations between BMI and global cognitive function after controlling for covariates (*p*s > 0.05).

### 5.5. Age and Gender as Moderators

Exploratory analyses were conducted to examine bidirectional associations between obesity (WHR and BMI) and cognitive function across age groups and gender. For age groups, participants were categorized as either younger (aged 30−45), middle−aged (aged 46−59), or older (above 59). Before conducting our exploratory analyses, a series of measurement invariance tests for both WHR and BMI models were conducted across time and age groups as well as across time and gender. Our analyses showed that the data fitted all the configural and metric invariance models well (RMSEAs < 0.08, CFIs > 0.90, TLIs > 0.90, and SRMRs < 0.08). However, our data only fitted well with scalar invariance models across time and gender for WHR (RMSEA = 0.065, CFI = 0.922., TLI = 0.908, and SRMR = 0.072) and BMI (RMSEA = 0.066, CFI = 0.931, TLI = 0.918, and SRMR = 0.073) but not across time and age groups for WHR (RMSEA = 0.074, CFI = 0.893., TLI = 0.875, and SRMR = 0.108) and BMI (RMSEA = 0.073, CFI = 0.907., TLI = 0.892, and SRMR = 0.107). Full invariance across time and age groups was supported in our configural and metric models (ΔCFI ≤ 0.005) but not in the scalar model (ΔCFI > 0.01). Similarly, full invariance across time and gender was supported in our configural and metrics models (ΔCFIs = 0.002) but not in the scalar model (ΔCFIs > 0.01). Inspection on our modification indices showed that majority of our tasks did not achieve scalar invariance across age groups and gender. Thus, for the purpose of our exploratory analyses, we only constrained factor structure and factor loadings in our subsequent multigroup cross-lagged panel models. 

To explore age groups and gender as moderators of the bidirectional relations between obesity and cognitive functions, a series of Wald tests of parameter constraints were conducted between the coefficients of our structural models with demographics, SES, and health covariates across age groups. We did not find any significant differences in our cross-lagged paths across age groups (*p*s > 0.10), except for the relations between WHR at Time 1 and executive function at Time 2 (Wald χ^2^ (2) = 10.47, *p* = 0.005). Specifically, WHR at Time 1 was significantly associated with executive function at Time 2 among younger (β = −0.077, *SE* = 0.035, *p* = 0.030) and middle-aged (β = −0.062, *SE* = 0.025, *p* = 0.011) but not older adults (β = −0.004, *SE* = 0.031, *p* = 0.887). Similarly, we did not find any differences in our cross-lagged paths across gender for both WHR and BMI (*p*s > 0.10).

## 6. Discussion

The popularity of BMI as a measure of obesity, overreliance on cross-sectional designs, and lack of specificity over the domains that underlie cognitive function contribute to mixed findings on the obesity–cognition link. To address these issues, we used data from the Cognitive Project of the MIDUS, a large-scale, longitudinal dataset on midlife adults, which allowed us to examine the long-term and bidirectional relations between obesity and two underlying domains of cognitive ability, namely executive function and episodic memory.

Our analyses unveiled several key findings. WHR at Time 1 was found to be significantly predictive of poorer episodic memory at Time 2 and this relationship held after controlling for demographic, SES, and health covariates. However, WHR at Time 1 did not predict changes in executive function at Time 2. Our results also showed that executive function at Time 1 was significantly associated with reduced WHR at Time 2 after controlling for potential confounds, but no relationship was found between episodic memory at Time 1 and WHR at Time 2. In contrast to the complex longitudinal associations documented between WHR and cognition, we failed to find any significant associations between BMI at Time 1 and latent cognitive factors at Time 2 as well as between latent cognitive factors at Time 1 and BMI at Time 2. In summary, our investigation based on midlife adults indicated that an increase in WHR is uniquely associated with episodic memory decline after nine years, while an increase in executive function is uniquely associated with reduced WHR over the same time span.

Consistent with previous research, we found WHR to be superior to BMI as a measure of the influence of obesity on cognition [14,15]. Our results therefore suggest that the use of BMI may obscure important associations between excess adiposity and cognitive function. Indeed, BMI does not distinguish between muscle and adipose tissue or directly assess regional adiposity [46], and WHR has also been found to correlate more strongly than BMI does with health risk factors including diabetes and various forms of cardiovascular disease [47,48,49]. As such, further research on obesity should rely less on BMI or at least utilize other measures in addition to BMI.

Second, we replicated previous research showing that WHR is distinctly associated with episodic memory beyond potential demographic, SES, and health confounds [15]. More importantly, WHR at Time 1 was not associated with global cognitive function. The result may suggest that the overall negative effect of WHR is domain-specific to episodic memory. This lends support to the view that obesity has an independent detrimental influence on encoding or retrieval processes. Some candidate mechanisms of poorer episodic memory due to the accumulation of visceral fat include the release of pro-inflammatory cytokines, which can lead to insulin resistance and aggravate cerebrovascular reactivity [50,51,52], and central and adipose inflammation, which may undermine synaptic plasticity [21,53]. However, in contrast to some previous studies, we did not find any evidence of long-term effects of obesity on executive functions [2], nor did we find evidence suggesting that poorer episodic memory increases the likelihood of obesity [16,18]. 

Our analyses revealed a nuanced relationship between obesity and cognitive ability. Specifically, we found that an increase in obesity is associated with decline in episodic memory but not executive function, and interestingly, we also found an increase in executive function to be associated with a reduction in obesity. Some preliminary evidence indeed suggests that executive function may have an impact on consumption and weight. For instance, deficits in executive function (e.g., attention-deficit/hyperactivity disorder) may foster dysregulated eating behaviors, such as binge eating or other forms of eating in the absence of hunger, or hamper regular and structured physical activity, which in turn may contribute to overeating and unsuccessful weight loss, thereby constituting a risk factor for obesity [54]. As these findings and suggestions are insightful but speculative, further research is warranted. Importantly, our results also highlight that the links between obesity and cognition are bidirectional. More attention should therefore be paid toward understanding their reciprocal dynamics.

In our multigroup analyses, we found significant associations between WHR at Time 1 and executive function at Time 2 among younger and middle-aged but not older adults. The results suggest that executive function may still be negatively implicated by WHR in specific age groups. These results are consistent with studies showing that weight gain is negatively associated with cognitive performance in relatively younger individuals, but this relationship is reversed for individuals with a mean age of above 73 [55,56]. As discussed by Smith and colleagues [57], two biological mechanisms may explain these findings. First, overweight men tend to retain more testosterone in their body fat, which may help to buffer against cognitive impairment from increased conversion to estrogen in old age [58]. Second, increased leptin due to higher adiposity in older adults may protect cognition [59]. However, this may be due to a survival effect in an elderly sample [57]. That is, obese middle-aged individuals are more likely to die at younger ages compared with individuals with elderly onset obesity, and those who did not die may have traits that increased survivability. 

Some limitations of the present research should be noted. First, the overall effect size of the longitudinal relationships between obesity and cognitive functions is small, which suggests that cognitive function may not be a significant factor in the prevention of obesity and raises the need to interpret the current findings with caution. Second, although we had longitudinal data and were able to rule out numerous confounding factors, our findings are essentially correlational, which limits our ability to make causal claims. Further research that employs controlled comparisons between groups of obese and non-obese individuals over multiple time points are warranted. By increasing the resolution of the bidirectional effects between cognition and weight, we can gain a better understanding of the protective and risk factors of weight on cognitive decline at specific ages or life stages. Furthermore, adiposity fat percentage [60] and diet have a demonstrable effect on cognition [17,61], but we were unable to include these factors as covariates due to the lack of such information in the MIDUS dataset. In sum, future investigations should strive to make systematic comparisons of cognitive ability between groups of individuals varying in weight while accounting for percentage of adiposity fat, dietary habits, and other potential confounds.

Taken together, the current investigation reveals a set of nuanced long-term relationships between obesity and episodic memory as well as obesity and executive function, and that these associations are best illuminated with WHR rather than BMI. As such, we highlight the need for future studies to consider the complex bidirectional relations between obesity and cognition and use more sensitive measures of obesity, in particular WHR with more carefully controlled studies.

## Figures and Tables

**Table 1:** Descriptive statistics of MIDUS II and MIDUS III samples.

	MIDUS II (2004–2006)		MIDUS III (2013–2017)	
	*M (SD)*	Range	*M (SD)*	Range
Mean Age (years)	55.01 (11.17)	33–83	64.10 (11.17)	42–92
Sex (% of Male)	45.8%		45.8%	
Education Attainment	7.58 (2.51)	1–12	7.61 (2.53)	1–12
Household Income (in $1000)	76.18 (62.50)	0–300	87.69 (72.52)	0–300
Smoking History (%)	45.2%		43.9%	
Alcohol Problem (%)	3.8%		7.5%	
Chronic Disease				
Diabetes (%)	7.6%		15.1%	
Hypertension (%)	25.8%		39.4%	
Stroke (%)	0.5%		1.4%	
Obesity				
Body Mass Index	28.02 (5.47)	18.56–50.66	28.37 (5.85)	18.51–52.52
Waist-to-Hip Ratio	0.91 (0.11)	0.50–1.48	9.24 (0.10)	0.52–1.38
Cognitive Functions				
Immediate Word List Recall	6.99 (2.18)	0–15	6.69 (2.36)	0–15
Backward Digit Span	5.09 (1.46)	0–8	4.97 (1.47)	0–8
Categorical Fluency	19.73 (5.99)	1–42	18.81 (6.06)	0–40
Delayed Word List Recall	4.68 (2.49)	0–14	4.37 (2.67)	0–14
Number Series	2.51 (1.50)	0–5	2.34 (1.56)	0–5
Backward Counting Task	38.69 (11.22)	−2–90	36.33 (11.52)	−10–90
Stop and Go Switch Task	1.07 (0.23)	0.61–3.77	1.27 (0.39)	0.42–7.67

Note: Mean (M) and standard deviation (SD); SDs are shown in parentheses. Education attainment was rated on a scale of 1 (No school) to 12 (Ph.D, ED. D, MD, LLB, LLD, JD, or other professional degree). Alcohol abuse was measured with a five-item modified Michigan Alcohol Screening Test (Selzer et al., 1975) [26]. Descriptive statistics were presented before imputation.

**Table 2:** Measurement invariance fit indices in waist-to-hip ratio and body mass index (BMI) across two waves.

Model	*χ^2^*	df	CFI	TLI	RMSEA	SRMR
**Waist-to-hip Ratio**						
Configural	427.547	84	0.977	0.968	0.039	0.039
Metric	443.910	89	0.977	0.969	0.039	0.040
Scalar	895.328	94	0.947	0.933	0.057	0.059
Partial Scalar	459.243	93	0.976	0.969	0.039	0.040
Unadjusted Cross-Lagged Panel	459.243	93	0.976	0.969	0.039	0.040
Cross-Lagged with Demographics and SES Covariates	718.116	133	0.968	0.954	0.041	0.037
Cross-Lagged Panel with Demographics, SES, and Health Covariates	774.519	183	0.968	0.953	0.035	0.031
**Body Mass Index**						
Configural	430.484	84	0.980	0.971	0.039	0.037
Metric	446.231	89	0.979	0.972	0.039	0.038
Scalar	899.458	94	0.952	0.939	0.057	0.058
Partial Scalar	462.120	93	0.978	0.972	0.039	0.039
Cross-Lagged	462.120	93	0.978	0.972	0.039	0.039
Cross-Lagged with Demographics and SES Covariates	727.138	133	0.969	0.956	0.041	0.036
Cross-Lagged Panel with Demographics, SES, and Health Covariates	784.558	183	0.970	0.956	0.035	0.030

Note: df = degree of freedom, CFI = comparative fit index, TLI = Tucker-Lewis index, RMSEA = root square error of approximation, SRMR = standardized root mean square residual.

**Table 3:** Standardized autoregressive and cross-lagged paths coefficients of obesity (waist-to-hip ratio and BMI) and the latent variables of executive function and episodic memory.

	Unadjusted Cross-Lagged Panel Model	Cross-Lagged Panel with Demographic and SES Covariates	Cross-Lagged Panel with Demographic, SES, and Health Covariates
**Waist-to-Hip Ratio**			
Autoregressive Paths			
WHR_T1_ → WHR_T2_	0.551 (0.023) **	0.420 (0.030) **	0.415 (0.030) **
EF_T1_ → EF_T2_	0.943 (0.011) **	0.890 (0.020) **	0.887 (0.020) **
EM_T1_ → EM_T2_	0.473 (0.024) **	0.409 (0.026) **	0.407 (0.026) **
Cross-Lagged Paths			
WHR_T1_ → EF_T2_	−0.024 (0.012) ^†^	−0.013 (0.014)	−0.010 (0.014)
WHR_T1_ → EM_T2_	−0.125 (0.017) **	−0.045 (0.019) *	−0.044 (0.019) *
EF_T1_ → WHR_T2_	0.009 (0.025)	−0.073 (0.034) **	−0.067 (0.033) *
EF_T1_ → EM_T2_	0.217 (0.024) **	0.179 (0.033) **	0.176 (0.033) **
EM_T1_ → WHR_T2_	−0.074 (0.023) *	−0.002 (0.024)	−0.002 (0.024)
EM_T1_ → EF_T2_	−0.002 (0.016)	−0.021 (0.017)	−0.022 (0.017)
**Body Mass Index**			
Autoregressive Paths			
BMI_T1_ → BMI_T2_	0.857 (0.009) **	0.850 (0.010) **	0.844 (0.011) **
EF_T1_ → EF_T2_	0.942 (0.011) **	0.891 (0.020) **	0.888 (0.020) **
EM_T1_ → EM_T2_	0.506 (0.023) **	0.410 (0.026) **	0.409 (0.026) **
Cross−Lagged Paths			
BMI_T1_ → EF_T2_	−0.002 (0.012)	−0.010 (0.012)	−0.007 (0.012)
BMI_T1_ → EM_T2_	0.015 (0.019)	0.007 (0.018)	0.005 (0.019)
EF_T1_ → BMI_T2_	0.021 (0.016)	−0.010 (0.022)	−0.010 (0.023)
EF_T1_ → EM_T2_	0.206 (0.024) **	0.183 (0.033) **	0.181 (0.033) **
EM_T1_ → BMI_T2_	0.030 (0.015) *	0.012 (0.016)	0.011 (0.016)
EM_T1_ → EF_T2_	−0.003 (0.016)	−0.021 (0.017)	−0.022 (0.017)

Note: Standardized errors shown in parentheses. EF = executive function, EM = episodic memory. ^†^ *p* < 0.10 * *p* < 0.05, ** *p* < 0.01.

**Table 4:** Measurement invariance fit indices in waist-to-hip ratio and BMI across two waves with cognitive function as a single latent factor.

Model	*χ^2^*	df	CFI	TLI	RMSEA	SRMR
**Waist-to-Hip Ratio**						
Configural	604.84	89	0.966	0.954	0.047	0.055
Metric	624.14	95	0.965	0.956	0.046	0.057
Scalar	1072.84	101	0.936	0.924	0.060	0.072
Partial Scalar	642.40	100	0.964	0.957	0.045	0.057
Unadjusted Cross−Lagged Panel	642.40	100	0.964	0.957	0.045	0.057
Cross−Lagged with Demographics and SES Covariates	1212.11	148	0.942	0.926	0.052	0.061
Cross−Lagged Panel with Demographics, SES, and Health Covariates	1294.09	208	0.941	0.925	0.044	0.050
**Body Mass Index**						
Configural	463.25	89	0.978	0.970	0.040	0.038
Metric	482.93	95	0.977	0.971	0.039	0.039
Scalar	937.41	101	0.951	0.941	0.056	0.059
Partial Scalar	500.78	100	0.976	0.972	0.039	0.040
Cross−Lagged	500.78	100	0.976	0.972	0.039	0.040
Cross−Lagged with Demographics and SES Covariates	1216.47	148	0.945	0.930	0.052	0.054
Cross−Lagged Panel with Demographics, SES, and Health Covariates	1299.22	208	0.945	0.930	0.044	0.045

Note: df = degree of freedom, CFI = comparative fit index, TLI = Tucker−Lewis index, RMSEA = root square error of approximation, SRMR = standardized root mean square residual.

**Table 5:** Standardized autoregressive and cross-lagged paths coefficients of obesity (waist-to-hip ratio and BMI) and the latent variables of global cognitive function.

	Unadjusted Cross-Lagged Panel Model	Cross-Lagged Panel with Demographic and SES Covariates	Cross-Lagged Panel with Demographic, SES, and Health Covariates
**Waist-to-Hip Ratio**			
Autoregressive Paths			
WHR_T1_ → WHR_T2_	0.563 (0.022) *	0.420 (0.030) **	0.415 (0.030) **
CF_T1_ → CF_T2_	0.933 (0.007) **	0.860 (0.016) **	0.856 (0.016) **
Cross−Lagged Paths			
WHR_T1_ → CF_T2_	−0.023 (0.012) ^†^	−0.016 (0.014)	−0.012 (0.014)
CF_T1_ → WHR_T2_	−0.032 (0.025)	−0.075 (0.030) *	−0.068 (0.030) *
**Body Mass Index**			
Autoregressive Paths			
BMI_T1_ → BMI_T2_	0.857 (0.009) **	0.849 (0.010) **	0.844 (0.011) **
CF_T1_ → CF_T2_	0.935 (0.007) **	0.862 (0.016) **	0.858 (0.016) **
Cross−Lagged Paths			
BMI_T1_ → CF_T2_	0.004 (0.012)	−0.005 (0.012)	−0.003 (0.012)
CF_T1_ → BMI_T2_	0.039 (0.014) *	−0.002 (0.019)	−0.002 (0.020)

Note: Standardized errors shown in parentheses. CF = cognitive function. ^†^ *p* < 0.10 * *p* < 0.05, ** *p* < 0.01.
